# Challenges in the control of neglected insect vector diseases of human importance in the Anglo-Caribbean

**DOI:** 10.1016/j.onehlt.2021.100316

**Published:** 2021-08-24

**Authors:** Sheena Francis, Chelsea Frank, Luke Buchanan, Sean Green, Roxann Stennett-Brown, Georgiana Gordon-Strachan, Yasmin Rubio-Palis, Charles Grant, Ruby Lisa Alexander-Lindo, Chukwuemeka Nwokocha, Dwight Robinson, Rupika Delgoda

**Affiliations:** a Natural Products Institute, University of the West Indies, Mona, Jamaica; b Mona Geoinformatics Institute, University of the West Indies, Mona, Jamaica; c Department of Life Sciences, University of the West Indies, Mona, Jamaica; d Department of Physics, University of the West Indies, Mona, Jamaica; e Caribbean Institute for Health Research, University of the West Indies, Mona, Jamaica; f Facultad de Ciencias de la Salud, sede Aragua, Universidad de Carabobo, Maracay, Venezuela; g International Centre for Environmental and Nuclear Sciences, University of the West Indies, Mona, Jamaica; h Department of Basic Medical Sciences, University of the West Indies, Mona, Jamaica; i Mosquito Control Research Unit, University of the West Indies, Jamaica

**Keywords:** Neglected tropical diseases, Tropical medicine, Infectious diseases, Arthropod-borne disease, Caribbean, Mosquito, Insect

## Abstract

**Introduction:**

Neglected tropical diseases (NTDs) in developing countries like the Caribbean, negatively affect multiple income-generating sectors, including the tourism industry upon which island states are highly dependent. Insect-transmitted NTDs include, but are not limited to, malaria, dengue and lymphatic filariasis. Control measures for these disease, are often ignored because of the associated cost. Many of the developing country members are thus retained in a financially crippling cycle, balancing the cost of prophylactic measures with that of controlling an outbreak.

The purpose of the paper is to bring awareness to NTDs transmitted by insects of importance to humans, and to assess factors affecting such control, in the English-speaking Caribbean.

**Method:**

Comprehensive literature review on reports pertaining to NTDs transmitted by insects in the Caribbean and Latin America was conducted. Data search was carried out on PubMed, and WHO and PAHO websites.

**Results and conclusion:**

Potential risk factors for NTDs transmitted by arthropods in the English-speaking Caribbean are summarised. The mosquito appears to be the main insect-vector of human importance within the region of concern. Arthropod-vectors of diseases of veterinary importance are also relevant because they affect the livelihood of farmers, in highly agriculture based economies. Other NTDs may also be in circulation gauged by the presence of antibodies in Caribbean individuals. However, routine diagnostic tests for specific diseases are expensive and tests may not be conducted when diseases are not prevalent in the population. It appears that only a few English-speaking Caribbean countries have examined secondary reservoirs of pathogens or assessed the effectivity of their insect control methods. As such, disease risk assessment appears incomplete. Although continuous control is financially demanding, an integrated and multisectoral approach might help to deflect the cost. Such interventions are now being promoted by health agencies within the region and various countries are creating and exploring the use of novel tools to be incorporated in their insect-vector control programmes.

## Highlights

• Neglected tropical disease transmitted (NTDs) by insects in English-speaking Caribbean
• Past and current challenges associated with the control of NTDs transmitted by insects in the English-speaking Caribbean.
• Potential risks factors of arthropod-borne diseases in the English-speaking Caribbean.
• Highlights on research areas that could strengthen elimination strategies of insect transmitted NTDs in the Caribbean.

## 1 Introduction

**Fig. 1:**
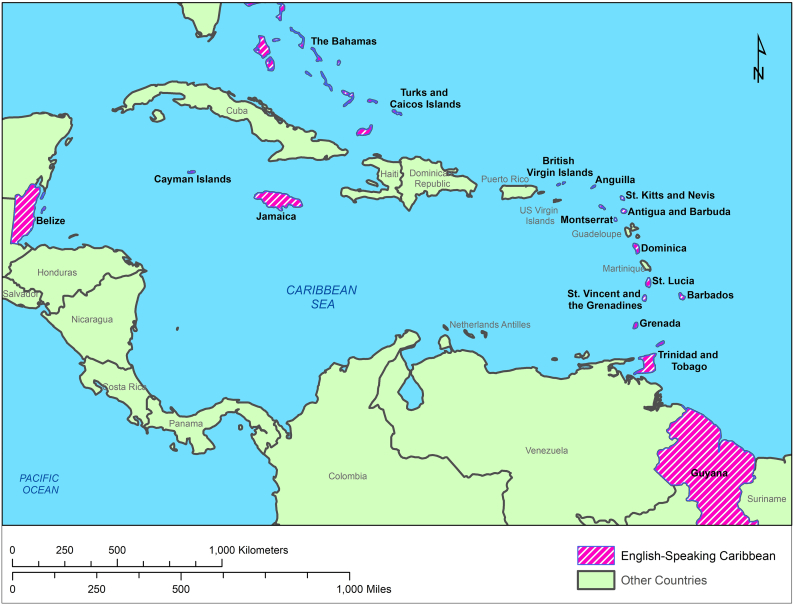
Map highlighting the English-Speaking Caribbean countries. (Map created using Arc-GIS using version 10.6) Highlighted in purple are countries within the Caribbean that are a part of the Commonwealth of Nations, inclusive of overseas territories – The islands – Anguilla, Antigua and Barbuda, The Bahamas, Barbados, British Virgin Islands, Cayman Islands, Dominica, Grenada, Jamaica, Monserrat, Turks and Caicos Islands, St. Kitts and Nevis, St. Lucia, St. Vincent and the Grenadines, Trinidad and Tobago. Continent Countries – Belize and Guyana. The Caribbean is comprised of several archipelagos- The Lucayan archipelago (The Bahamas and the Turks and Caicos islands), the Greater Antilles (Cuba, Hispaniola, Jamaica, Puerto Rico and the Cayman Islands), the Lesser Antilles (made up of the arc islands from the Virgin Islands to the Trinidad), and the Leeward Antilles (Aruba, Bonaire and Curaçao).

Neglected tropical diseases (NTD) transmitted by hematophagous arthropods that are of human importance, include but are not limited to, dengue, chikungunya, malaria, filariasis, onchocerciasis, leishmaniasis, and chagas disease. Insect transmitted NTDs are usually endemic in developing countries [1], such as many in the Caribbean, and are problematic in low-income communities, particularly where water availability and waste management are poor. Often, the cost of management of vectors and prevention of disease, pose serious challenges for developing countries and as such, the systematic control of insect transmitted NTDs remain elusive, until an outbreak forces a temporary, band-aid approach to salvage some control [[1], [2], [3]]. Spotlights have been shone on major outbreaks in the Caribbean, due its geographic importance as a gateway to North and South America, and it is clear that management of these outbreaks in these islands is of significant value to the wider region. This paper therefore aims to bring into sharp focus, key parameters involved in the management of insect transmitted NTDs in the English-speaking Caribbean (Fig. 1).

The review firstly presents insect-borne diseases of importance to humans, reported in the Caribbean, and known underlying causative agents - insect vectors, followed by challenges in insect-vector management, with a brief highlight on important/developing threats, as well as challenges posed by climate change, given the particular sensitivity of the region to this global phenomenon. Vector monitoring and surveillance tools available in the region along with some challenges associated with their use are also presented.

Although multiple studies exist on arthropod-borne diseases in the Americas, entomological and epidemiological publications examining the presence of arthropods and their pathogens within the English-speaking Caribbean, are very limited [4,5]. This review highlights the presence of insect-borne diseases reported in the Caribbean, with an aim to bring about awareness to the region, specifically in the English-speaking Caribbean (Fig. 1), where information appears lacking. The review hopes to bridge knowledge gaps that could promote awareness and a heightened vigilance for future outbreaks.

## 2 The Caribbean and its neglected tropical diseases

### 2.1 Situational analysis

The geographic location and climate of the Caribbean archipelagos (Fig. 1) present suitable habitats for many pathogenic arthropods that thrive in warm, moist climate. The Karst and mountain topography of the islands that forms rock holes, as well as the rich vegetation, where tree stumps, tree holes, leaf axils and fruit pericarp, all provide unique habitats for insect proliferation [6]. The Caribbean region is especially vulnerable to insect-vector borne diseases, which have devastating impact on the health, economy and social fabric of these small island developing states (SIDS). The Caribbean relies heavily on tourism to balance its fiscal budget. The limited medications to treat the diseases, the high risk of rapid transmission through travel, the risk of infection and the associated negative publicity, collectively result in decreased tourist travel during disease outbreaks [2,7,8]. Reduced labour force during disease outbreaks leads to reduced productivity and low earning potential within the region [8]. The underlying consequence of these economic and environmental conditions coupled with the looming impact of climate change [9] in the region and the potential threat of non-arboviruses presents a potent package of unknown risk to the region.

Summarised in Table 1, Table 2 are the insect-vectors of the Caribbean that transmit NTDs that have been either detected or suspected in humans living within the Caribbean. Some insect-vectors appear localised to a region of the Caribbean, for example *Triatoma dimidiate,* an insect found in the forested areas of Belize and neighbouring Latin American countries, and usually transmits *Trypanosoma cruzi* to forest dwelling animals [10]. Some pathogens within the Caribbean appear to have region specific vectors, an example of such is, *Mansonella ozzardi*, which causes Mansonelliasis. The disease has been well discussed [[11], [12], [13], [14]], though it is considered as a forgotten insect-vector borne disease, that is neglected by health authorities in some countries [14]; possibly because infections are generally mild to asymptomatic, and go undetected. Throughout the Caribbean, the insect-vector that transmit *Mansonella ozzardi* are *Culicoides* spp*.*, however, along the Amazon border of Guyana and southern Latin America countries, the pathogen is transmitted mainly by *Simulium* spp. [11]. More common insect-vectors and pathogens of interests, based on unique cases, increased detection of pathogens in forest insects or observation in novel locations are described.

**Table 1:** Non-mosquito insect-borne diseases observed in the Caribbean and Latin America.

Insect Vector (s) Type, Family, Genus species	Associated Pathogen	Disease caused by Arbovirus	Reported Pathogen Reservoir	Detection in Humans in Latin American Countries	Detection in Humans from Non-Hispanic Caribbean Countries	Common Treatments	Reference
Louse *Pediculus humanus*	(a) *Rickettsia prowazekii* (b) *Bartonella quintana* (*Rocalimaea quintana)* (c) *Borrelia recurrentis* *(d) Rickettsia typhi* (Rickettsia mooseri)	-(a and d) Typhus -(b) Trench (Quintana) fever -(c) louse-borne relapsing fever	-(a) Louse and Flying squirrels -(b and c) Louse humans -(d) Rats	Yes	(d) Low seroprevalence (< 2%) for the *Rickettsia typhi* antibody were among test population. No Caribbean studies are available that definitively links the transmission of the pathogen to lice.	antibiotics	[16,28]

Phlebotomine Sandflies *#Lutzomyia spp*.	*Leishmania spp.*	Leishmaniases Exist in three forms (i) Visceral (ii) cutaneous (iii) mucocutaneous	-Humans -Rats -Mongoose, −Marsupials -Dogs	Yes	Sporadic cases of cutaneous form. In Martinique, 1 case study identified mucocutaneous form. Cutaneous form endemic in Belize and Guyana	Pentavalent antimonial (Drug resistance reported) vector control necessary	[16,22,23,89]

Flea *Xenopsylla cheopis*	(a) ^*Yersinia spp*. (b) *Rickettsia typhi* (Rickettsia mooseri)	(a) Plague or enteric maladies (b) Rickettsiosis	-Rodents -Monkeys -Humans	Yes	*-* Low seroprevalence (< 2%) for the *Rickettsia typhi* antibody were among test population. Possibly transmitted by rat flea.	antibiotics	[16,27,28,67]

Midges *§Culicoides* spp. *Culicoides furens* *Culicoides barbosai* (are important in Haiti) *Culicoides phlebotomus* (are important in Trinidad) *(Simuliidae are responsible for Latin American transmission)*	*Mansonella ozzardi*	Mansonelliasis	Human	Yes Studies have shown that repeated bites necessary to produce infection	Detected in -Haiti	Ivermectin	[14]

Blackflies *Simuliidae* *Simulium spp.*	*Onchocerca volvulus*	Onchocerciasis (river blindness)	Human	Yes Studies have shown that repeated bites necessary to produce infection	All Caribbean countries are classified as non-endemic. No evidence of human infection in the region could be found.	Ivermectin	[12,16,89,90]

Triatomine bugs *Panstrongylus spp.* *Rhodnuis spp.* *Triatoma spp.* #Triatominae (cockroaches and houseflies can act as carriers)	^*Trypanosoma cruzi*	Chagas disease	-Dog, -Sheep -Rats -Cotton-tailed Rabbits, -Human -Marsupials -Blood transfusion	Yes Endemic areas of 21 countries	Endemic in Guyana. Serological studies conducted in the 1960s, in humans from Jamaica &Trinidad showing cardiac myopathies, were positive for the antibody. Feral animals from Grenada, Aruba & Brazil tested positive for the antibodies (studies conducted in the 1960s), but negative in humans from those countries. Serological studies found 0.004% of 888 natives from Belize were positive for the *T. cruzi* antibody; The *Triatoma dimidiata*, a sylvatic vector is sometimes found in and around human dwellings. These bugs may the vector of epidemiological importance for Chagas in Belize.	benznidazole or nifurtimox Dependent on disease manifestation Vector control via insecticides remains cost effective	[16,29,30,32]

The table shows insect vectors in the region, their pathogens, reported pathogen reservoirs in the Caribbean, and medications normally used to combat the disease.

Table Key: # Vector specific to the Caribbean and Latin America, ^ viruses having Sylvatic cycle, § vectors responsible for Caribbean transmission.

**Table 2:** Mosquito-borne diseases observed in the Caribbean and Latin America.

Mosquitoes of the Caribbean Genus species	Associated Pathogens Order/ family, genus, spp.	Disease caused by Arbovirus	Reported Pathogen Reservoir	Detection in Humans in Latin American Countries	Detection in Humans in Non-Hispanic Caribbean Countries	Common Treatments	Reference
*-A. aegypti* *-Aedes albopictus*	*Togaviridae* *alphavirus* *Chikungunya virus*	Chikungunya	Possibly Bats (Grenada)	Yes	2013, first appearance in St. Martin. The disease is considered endemic to the Region	analgesic & NSAIDs	[16,71]

*-Mansonia venezuelensis* Mainly found in forested or rural areas *-Haemagogus janthinomys* Mainly found in forested or rural areas *-A. aegypti* *-Aedes albopictus*	*Togaviridae* *alphavirus* *Mayaro virus*	Mayaro		Yes	First isolated in Trinidad in 1954, since then rare occurrences have been reported in very rural areas. Detected in Haiti in 2016.	Symptomatic treatment	[16,73,91]

*-A. aegypti* *-Aedes albopictus*	*Flaviviridae* *Flavivirus* *Yellow fever virus*	Urban Yellow Fever	Man	Yes	Detected prior to the 17th century. Since then, it has been successfully eradicated in Cuba 1900 and other small islands However, sporadic cases have been reported Guyana Trinidad and Tobago Surinam French Guiana	Vaccine	[16,92]

*- Haemagogus janthinomys* Mainly found in forested or rural areas *-Sabethes spp.* Mainly found in forested or rural areas	*Flaviviridae* *Flavivirus* ^*Yellow fever virus*	Sylvan (Jungle) yellow fever	-Mammals: wild Monkeys belonging the Cercopithecidae, Colobidae and Cebidae Family, The Galogao senegalensis	Yes	Isolated cases have been found in Trinidad & French Guiana	Vaccine	[16,[93], [94], [95]]

*-A. aegypti* *-Aedes albopictus* *-Aedes mediovittatus*	*Flaviviridae* *Flavivirus* ^*Dengue virus*	Dengue	-Aedes spp. (transovarial) -Human -Sylvatic cycle (monkeys and canopy mosquitoes)	Yes	Initially observed in: 1953 DENV2 – Trinidad 1963 DENV3 – Puerto Rico 1977 DENV1 – Multiple islands 1981 DENV4 – multiple islands The disease is considered endemic to the Region.	Treatment dependent on the severity	[16,96]

*-A. aegypti* *-Aedes albopictus*	*Flaviviridae* *Flavivirus* ^*Zika virus*	Zika		Yes	Initially observed in 2015, The disease is considered endemic to the Region	analgesic & NSAIDs	[16]

*-Culex spp.* *-(Argasidae Ticks)*	*Flaviviridae* *Flavivirus* *(a) St. Louis encephalitis virus* *(b) West Nile virus*	(a) St. Louis encephalitis (b) West Nile Fever	-Birds -Domesticated animals	Yes	During an outbreak around 2001, serological studies detected antibodies to West Nile Virus in humans in Cayman, Bahamas, Haiti	Treatment depends on severity	[16,97]

*(a)- Coquillettidia venezuelensis* Mainly found in forested or rural areas *- Aedes serratus* *- Culex quinquefaciatus* *(-Culicoides paraensis* Midge*)* *(b)-Aedes scapularis*	*Order:* *Bunyaviridae* *Orthobunyavirus* ^(a) *Oropouche virus* ^(b) *Cache Valley orthobunyavirus*	(a) Oropouche fever (b) Cache Valley	(a)-Sloth -Cebus monkey -Howler monkey -Marsupial -Birds (b) -Mice -Horse -Monkey(Alouatta)	Yes -Peru -Brazil -Panama	(a)Initially isolated in Trinidad in 1955, since then rare occurrences have been reported in very rural areas. (b)In 1958, antibodies were found in 15/46 human sera from Trinidad and 8/26 from Guyana, rare detections has occurred since its discovery.	Symptomatic treatment	[51,52,72,98]

*-An. darlingi* *-An. aquasalis* *-An freborni* *- An. quadrimaculatus* *-An. albimanus* *- An. nuneztovari* *-An pseudopunctipennis* *-An. argyritarsis* *-An. crucians*	*Plasmodiidae* *-Plasmodium vivax* *-Plasmodium falciparum;* Are most common, however, few cases have been caused by *P. ovale, P. malaria,* and *P. knowlesi*	Malaria	non-zoonotic	Yes	Detected prior to the 17th century. Cases within the past 20 years occur in Guyana Surinam French Guiana Haiti Dominica Republic Belize -However the disease has been eradicated in the islands except Hispaniola.	antimalarial drugs e.g. Chloroquine	[16]

*-Anopholes spp* *-Culex quinquefasciatus* *-Culex pipens* *-Culex spp.* *- Aedes spp* *-Masonia spp.* Mainly found in forested or rural areas	Nematodes *Onchocercidae* *-Wucheria banocrofti*	Lymphatic filariasis	-Domestic cats - Macaca -Monkeys	Yes	Detected prior to the 17th century. Cases within the past 20 years occur in Haiti Dominica Republic Guyana The diseases does not occur on the smaller islands. Recently classified non-endemic are Trinidad and Tobago & Surinam by WHO. Guyana is currently using the mass drug administration for the elimination of nematodes transmitted by mosquitoes.	Combined treatment of albendazole, ivermectin and diethylcarbamazine citrate	[16,89,90]

The table shows mosquito vectors in the region, their pathogens, reported pathogen reservoirs in the Caribbean, and medications normally used to combat the disease.

Table Key: ^ viruses having Sylvatic cycle; organisms within the bracket shows possible non-mosquito vectors of the disease.

### 2.2 Common insect vectors and their associated diseases in the Caribbean

#### 2.2.1 Sandflies

Phlebotomine sand flies are responsible for the transmission *Leishmania* spp*.,* a protozoa species that causes Leishmaniasis. The disease has three main forms- cutaneous, the most common, mucocutaneous, and visceral, the most severe. The pathogen and its vector are widely distributed in impoverished communities globally, where it thrives in immunocompromised individuals [15]. Over 1-million new cases are reported yearly, worldwide, resulting in 20,000 deaths annually [16]. Cases of cutaneous leishmaniasis have been reported throughout the Caribbean, including in Martinique, Guadeloupe, Trinidad [[15], [16], [17], [18]], Dominica Republic [19], Belize and Guyana [16]. Successful control and elimination programmes in the region have resulted in a reduction in the number of reported cases in endemic countries targeted to eliminate the disease by 2030. In 2019, Guyana, a country where cutaneous leishmaniasis is endemic, reported a 73% reduction in the number of incidences [20], owing to the mass distribution of therapeutics. The pathogenesis of the diseases appears to be temperature sensitive. Temperature sensitive pathogens generally remain within the cutaneous membrane and more temperature resistant strains migrate towards the viscera [21] of the individual. Recently, *L. martiniquensis,* a cutaneous species restricted to the island of Martinique, was observed for the first time to clinically manifest as visceral leishmaniasis in an individual previously diagnosed with the immunodeficient disease HIV [22] (Table 1). It appears that a shift in climate patterns is driving vector dispersal and mutagenesis in the pathogen, causing *Leishmania* spp. to occur in novel locations [23,24]. As temperature patterns continue to change within the region, sandflies and the mutagenesis of *Leishmania* spp. needs to be monitored.

#### 2.2.2 Fleas

Illnesses contributed by fleas within the Caribbean appear low*.* In light of symptoms caused by flea-borne diseases, which range from gastroenteritis to the plague, test for the pathogen it transmits, appears to be conducted routinely in the region during diarrheal outbreaks [25,26]. It appears that the only mammalian positive test of flea-disease pathogen, *Yersinia* sp., in the English Caribbean, is linked to a unique case in St. Kitts in 2012. Caged *Chlorocebus aethiops sabaeus* monkeys, displayed maladies associated with enteric disease. Splenic screens for the animals were positive for the specific mammalian *Yersinia* spp. antibodies [27]. Studies conducted in Trinidad on 423 diarrhoeic and non-diarrhoeic livestock from 50 farms showed *Yersinia* spp., contributed to 0.7% of the enteric bacterial infection [26]. These results suggest a low presence of *Yersinia* spp., pathogen in the region, and to date have not contributed to human enteric diseases in the region. Other research further supports the low impact of flea-borne diseases in the region. Studies conducted on pregnant women from the Caribbean showed low seroprevalence <2%, for the antibodies to typhus group rickettsiae [28], another flea-borne disease. The study was conducted across ten Caribbean countries, and only 6 countries had candidates that tested positive. The study suggests that *Rickettsia typhi,* a pathogen transmitted by murine flea, *Xenopsylla* spp., is not a common disease agent in the region.

#### 2.2.3 Triatomine bug

Chagas or American Trypanosomiasis is a disease caused by the parasite *Trypanosoma cruzi*, a pathogen restricted to the Americas. Triatomine bug are hematophagous insects that defecate during feeding. Their faecal matter contains multiple infectious protozoa, trypomastigotes, which then enters the body through breaks in the cutaneous membrane [16]. Trypanosomiasis is endemic in French Guiana, Guyana and Suriname [16], and evidence of its vectors and pathogen [10,29] occur throughout the Caribbean. However most epidemiological reports on chagas primarily originate in Latin America and Caribbean countries in close proximity to the Latin American continent (Table 1, Fig. 1)*.* Studies in the 1960s, found <10% of *Triatoma maculata* collected from Curaçao and Aruba [29] to be infected with *T. cruzi*. However, Feral animals (dogs, sheep, rats, rabbits) tested in Aruba showed antibodies against *T. cruzi*, but sera collected from over 2000 residents tested, were negative [30]. Other studies conducted around that period in patients displaying cardiomyopathies from Trinidad and Jamaica, were positive for antibodies against *T. cruzi* [10]. In the recent decade, serological studies conducted on stray (13%) and kept (6%) dogs in Grenada [31] as well as humans working or living in close proximity to forested areas of Belize (6.1%) showed evidence of exposure to *T. cruzi* [32]. It is speculated that human-*T. cruzi* exposure in the Caribbean is owed to the interaction of humans with infected forest dwelling Triatomine bugs [10]; animal reservoirs [31]; blood transfusion; or the movement of infected human immigrants into non-endemic countries [32].

#### 2.2.4 Mosquitoes

**Fig. 2:**
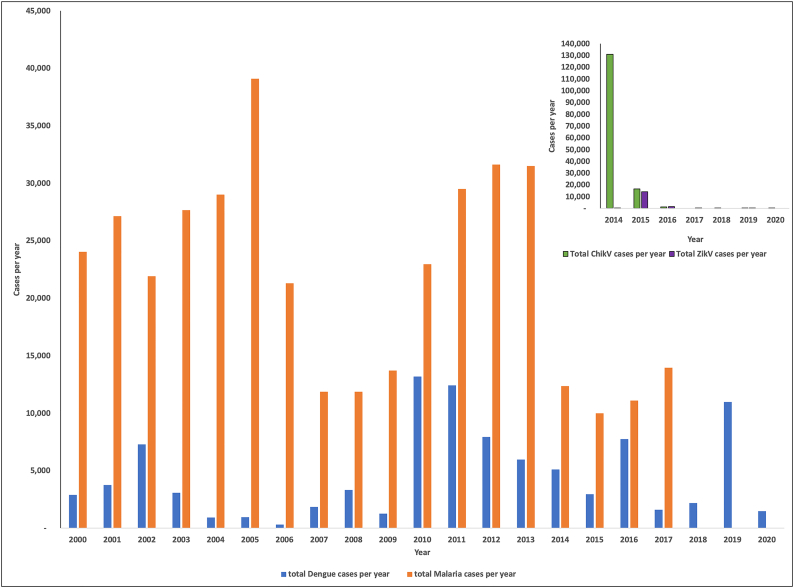
Graph showing reported cases of Insect transmitted NTDs in the English-speaking Caribbean of the past 20 years. Information obtained from PLISA [33]. Guyana, where malaria is endemic, is the main contributor to the total malaria cases reported throughout the English-speaking Caribbean. The majority of the malaria cases observed in the other English-speaking Caribbean countries appear to result from imported cases. Imported cases have resulted in outbreaks. The 2004–2007 outbreak in Jamaica, contributed to the high incidences of malaria observed within that period . Dengue on the hand, occur yearly in all English-speaking Caribbean countries. The insert graph shows cases reported for chikungunya (ChikV) and Zika (ZikV) viruses. The first viral transmission of these virus within the English-speaking Caribbean occurred between 2014 and 2015 respectively.

With the exception of continental English-speaking Caribbean countries, such as Guyana and Belize, and islands in close proximity to the Continent such as Trinidad and Tobago, the main arthropod vector of importance in the English-speaking Caribbean appears to be the mosquito. Each year regional data on mosquito-borne illnesses are pusblished [33]. Mosquito-borne illnesses in the Caribbean are often caused by a *flavivirus*, example, the dengue virus, which is transmitted by *Aedes* spp., or by a parasite, example, malaria causing *Plasmodium* spp., which is transmitted by *Anopheles* spp., (Fig. 2). However, other mosquito pathogens too, such as *Orthobunyavirus*, have been isolated in English-speaking Caribbean countries close to the Latin American continent (Table 2). Shown in Fig. 2, are the number of cases of the major mosquito-borne diseases, dengue, malaria, chikungunya and the Zika virus reported in the English Caribbean region between 2000 and 2020.

The control and prevention of mosquito-borne diseases started shortly after the discovery of their transmission vectors [34]. In the 1900s, organised health and sanitation programs involving the World Health Organization (WHO), local health authorities, the Pan American Sanitary Bureau (PASB) and the use of dichlorodiphenyltrichloroethane (DDT) led to the successful reduction of mosquito populations including the eradication of the *A. aegypti,* throughout the islands of the Caribbean. Further, the yellow fever and malaria pathogens, were eradicated within the Caribbean except in Hispaniola [34,35]. Although malaria on the smaller islands was successfully eradicated, periodic outbreaks results from imported cases. Such outbreaks are usually managed by the health authorities through the use of pesticides and the distribution of the anti-plasmodium drugs [35,36].

The *A. aegypti* mosquito, on the other hand, repopulated many countries from the 1970's onwards, resulting in sporadic observances in dengue fever (Fig. 2). However, in 2013, the vectoring potential of the *A. aegypti* mosquito, with its capability to transmit various pathogens became relevant to the Caribbean and Latin America. Chikungunya, an *alphavirus* transmitted by the *Aedes* spp., was previously localised to Asian and African countries. The first autochthonous case in the Western Hemisphere was reported in late 2013 in the Caribbean island, St. Martin. The virus, which causes debilitating arthritic-like symptoms rapidly spread throughout the Americas by the end of 2014 [37,38]. The Zika virus, a *flavivirus,* usually observed in Asian countries followed shortly in 2015 [38]. Not only was the virus transmitted by the *Aedes* spp., to humans, but also from humans to humans. The virus was found to be sexually transmitted and more importantly, observed to be transmitted vertically from mother to foetus, causing microcephaly in new-borns [39]. The long-term implications of both diseases are unknown. However continuous health care for new-borns infected with the Zika virus and its associated birth defects, is certain. Further, in 2019, the combined region of the Caribbean and Latin America reported the highest occurrence of dengue worldwide for the first time, despite the vigilance of the healthcare authorities [40]. These observations in the recent decade, highlights the need to assess potential risks of mosquito-borne diseases, to strengthen vector surveillance activities to effectively combat current and future healthcare challenges. More so, studies in the region have shown increases in competent species, invasive and native, established close to human dwellings [41,42] and insecticides losing their potency (Table 3) [[43], [44], [45]].

**Table 3:** The table showing a summary of reported Insecticide resistance in *A. aegypti* in the Caribbean.

Insecticide	Evidence of Insecticide Resistance 1980–1990	Evidence of Insecticide Resistance 2000–2020	*kdr* mutation observed V1016I/F1534C	Elevate Metabolic Enzymes Activity	Reference
**Organophosphates** Chlorpyrifos (C) Fenitrothion (F) Malathion (M) Temephos (T)	(All organophosphates listed) Antigua Bahamas Cuba Dominica Republic Dominica Grenada Jamaica Monserrat Puerto Rico St. Kitts & Nevis St. Lucia St. Vincent & Grenadines Trinidad British West Indies	(Malathion and Temephos) Grand Cayman Guadeloupe, Jamaica St. Martin			[65,99]

**Pyrethroids** Deltamethrin (D) Lambda cyhalothrin (L) Permethrin (P)	Dominica republic	Guadeloupe (D) Jamaica (D, L,P) St. Martin (D) Grand Cayman (D, L, P)	Guadeloupe, Jamaica St. Martin Grand Cayman	Jamaica Grand Cayman	[43,65,99]

**Organochlorides** DDT	Dominica Republic Jamaica	Grand Cayman	Grand Cayman	Grand Cayman	[99,100]

**Carbamates** Bendiocarb (B) Propoxur (Pr)	Dominica Republic (Pr) Grenada (Pr), Monserrat (Pr), St. Kitts & Nevis (Pr)	Jamaica (Pr, B)			[45,101]

Other mosquito-borne viruses that affect humans living in the Caribbean, though rarely, are the *Mayaro virus* and the *Oropouche virus* (Table 2)*.* The *Mayaro virus*, an *alphavirus,* first observed in four forest workers from Trinidad [46], affects individuals working or dwelling in forested areas in Trinidad [46], French Guiana [47], Brazil, and neighbouring countries, including Suriname. The outbreaks appear small and localised, and usually occur simultaneously with sylvatic yellow fever [48]. The vectors of the virus include forest mosquitoes [49](see Table 2 for species), as well as the anthropophilic *Aedes* mosquitoes. The non-specificity of vector facilitates movement of the virus from forested to urban areas with the aid of infected humans [50] or birds [48].

Oropouche fever caused by the *Oropouche virus* (*Orthobunyavirus* genus) is a febrile infection transmitted by *Coquillettidia venezuelensis* mosquitoes and sanguineous midges [51]. Ever since its initial isolation from humans and forest monkeys in Trinidad [52], the virus is the second causative agent of human illnesses in Brazil and Peru [53,54]. The outbreaks are usually explosive. The virus has been shown to cause infections to the central nervous system in immunocompromised individuals [55]. The virus is usually maintained in a sylvatic cycle [52].

### 2.3 Non-insect arthropods of importance in the Anglo-Caribbean

Although not an insect, Ticks, are worth mentioning in this review, as they are arthropod-vectors of economic significance in the region, that affect the health of live-stocks, and also infect humans [56]. The *Amblyomma variegatum*, widely distributed in the region, is thought to have arrived in the Caribbean during the 18th Century, with the introduction of cattle from Senegal and its dispersal facilitated either by regional trade of live-stock or by the migration of cattle egrets [57]. *Amblyomma variegatum* are vectors of bacterial *Rickettsia africae* and viral *Nairovirus* which causes African Spotted Fever and Crimean-Congo Hemorrhagic Fever virus in humans, respectively [56]. Serological studies on individuals living in the English Caribbean were positive, 33.5 ± 4.4%, for spotted fever group rickettsia [28]. The study demonstrated that African tick-borne fever may be common in the region. Studies conducted in select Caribbean countries, have shown *A. variegatum* to be the main vector of rickettsia [58,59]. The interaction of humans/farmers with animals infested with infected ticks, may facilitate tick-human interactions and the transmission of tick-borne disease to individuals in the region. Live-stock farming is an important sector in the Caribbean [60], as such, countries in the region have undertaken several tick eradication projects to improve animal health and to reduce economic losses incurred from tick-borne diseases [4]. Considering the limited success of the tick-eradication projects [4,58] and the results of the serology study for spotted fever group rickettsia, previously mentioned [56], investigations on the impact of ticks on human health in the region are relevant. Other tickborne diseases, example *Ehrlichia* spp*.*, transmitted by *Rhipicephalus* spp*.*, have been isolated from dogs (9% of 29 dogs) in the Caribbean [61], suggesting that Caribbean individuals could also be at risk for *Ehrlichia* spp., which clinically manifests as Human Monocytotropic Ehrlichiosis [62,63]. For a comprehensive review on ticks and tick-borne pathogens observed in the region see [4].

### 2.4 Challenges in insect-borne control in the region

#### 2.4.1 Consistency of vector management in light of knowledge gaps and limited resources

As previously mentioned, the English-speaking Caribbean countries have conducted several eradication programmes for nuisance insect vectors and their diseases. The lack of consistency and the overzealous use of insecticides have led to resistance and reinfestation [58,64,65]. The persistent climate and environmental challenges faced by many SIDS like the Caribbean, forces their government to prioritise spending [66]. The possibility of prioritising NTDs in terms of their economic impact on the country may underpin the practice of how funds are allocated to maintain prophylactic management to prevent reoccurrence after elimination. This would primarily be due to the realistic earning and spending potentials of the country.

Caribbean islands, except for Hispaniola and Trinidad have less arthropod-borne diseases in comparison to the continental Caribbean countries. This is possibly the result of the size of the islands and their limited forest range, which creates less vector niche in comparison to the continental countries [67]. As such, vector control programmes may be easier to managed on small islands in comparison to larger land mass [34].

Arthropod-borne diseases are transmitted to humans by either peridomestic (urban cycle) or canopy-dwelling (sylvatic cycle) vectors [68], or in some cases, through blood transfusion [32,64]. The increase in population size with human encroachment on forested areas [69] facilitates sylvatic vector-pathogen-human interactions [70], which can drive the spread of arthropod-borne diseases. Little is known about pathogenic reservoirs of arthropod-borne diseases within the region [71]; this is an area of research that requires immediate attention to fully comprehend potential risk factors of forest encroachment. Serological studies conducted on fruit bats, trapped one year following the introduction of chikungunya in Grenada showed that 15/42 bats were seropositive for the virus [71]. The study appears to be the only investigation of sylvatic viral reservoirs for chikungunya in the region.

*Mayaro* and *Oropouche* viruses, which are mainly transmitted by forest mosquitoes, are gaining concerns within the region because of the similarities in their symptoms to the more common mosquito-borne illnesses, such as dengue [47,68], making diagnosis difficult [72]. Additionally, mutated strains of the viruses have been found in new locations within the region. In 2015, the *Mayaro* virus was isolated from a child living in Haiti [73], the first report of the virus in the northern Caribbean. The case report demonstrated an increase in the geographical range of the virus as well as viral mutation. Prior to the report, *Mayaro* virus detection occurred only in the Southern basin of the Caribbean and Latin America. The phylogenetic profile of the *Mayaro* virus now, clearly shows two distinct clades, 1) a South American, which includes strains from Peru, Bolivia, Venezuela, Trinidad and Tobago, and French Guiana, and 2) the Haitian strain [73]. *The Oropouche* virus on the other hand, commonly displays genetic reassortment that leads to novel viruses, such as Iquitos and Madre de Dios viruses [54,72], has been maintained to date in southern Caribbean and Latin American countries, though increased pathogenicity of the virus appears common in nature.

From this review, it appears that few Caribbean countries routinely conduct epidemiological studies on pathogens of insect-vectors or reservoirs of insects-borne diseases. The knowledge of existing pathogens of arthropods in circulation is essential to determine prevalence, mutations or aberrant behaviour in pathogen and or vector.

The non-arbovirus pandemic- COVID-19, currently being observed in 2020–2021, further tests the mettle of the health sectors in the Caribbean. These countries are not only grappling with the impact of COVID-19, but the co-circulation of arboviruses, such as the dengue virus [75,76]. Latin American countries have reported malaria and dengue co-circulating with COVID-19 [77]. Funding and resources for vector control are typically quite limited in most Caribbean countries and Vector Control departments are usually understaffed. The management of the COVID-19 pandemic within the Americas, aggravates healthcare systems by weakening their limited resources. While the use of laboratory diagnostic technologies to properly identify circulating pathogens has been readily accepted by the health sectors, such technologies are still expensive and not sustainable for most countries on allocated budgets. Less prevalent pathogens co-circulating with highly prevalent pathogens that share similar symptoms, may go undetected [74] owing to the inaccessibility of epidemiological diagnostic tools.

#### 2.4.2 Climate impact on the incidences of arboviruses

Many studies have indicated correlation of insect transmitted NTDs with climate variables such as temperature and precipitation. Climatic variables have been shown to affect the development and proliferation of pathogens and arthropod-vectors, as well as the vectors ability to transmit diseases [78,79]. In the Caribbean, temporal correlation between dengue incidence and rainfall or temperature [80] have been reported. Studies in other parts of the world have associated epidemic outbreaks and the transmission of the disease with climate variability [81] (evidenced by the occurrences of El Niño Southern Oscillation/ENSO events) through temperature increases and availability of stagnant and stored mosquito-accessible water during droughts as well as after rains [78,79].

Rises in temperature and moisture content, seem to enhance dengue transmission rates as moisture is needed for the subsequent transformation of eggs to adults. Time to transformation appears to decrease with rise in temperature. An excess or lack of rain can contribute to an increase in breeding sites and vector productivity. Excess of rainfall results in pools of stagnant water on the ground or discarded containers, can become breeding sites. On the other hand, lack of rainfall encourages water storage in containers by community dwellers. These also can become potential breeding sites especially if the containers are not properly covered [78,79]. Additionally, in warmer climates blood meals are digested faster and adult female insects feed more frequently, increasing the transmission intensity. The development cycle of pathogens also appears reduced in warmer climate [23,24], thus infection rates can increase with increased temperature.

Other cases demonstrating change in arthropod-borne disease manifestation with increase temperature within the region is exemplified in *Leishmania* spp., which has been previously discussed [22].

## 3 Surveillance system, availability of tools and innovative vector control approaches in the Caribbean

#### 3.1.1 Entomological and epidemiological surveillance facilities and tools

Within the Caribbean the surveillance of arthropod-borne diseases takes place at different levels in the national health systems of each country (district, parish, regional and national). Syndromic surveillance and country disease data on endemic mosquito-borne diseases, such as dengue, and other circulating arboviruses are collated and reported on a regular basis to the Caribbean Public Health Agency (CARPHA), which collates and analyses public health surveillance data at the regional level [82]. The CARPHA Medical Microbiology laboratory (CMML) also offers molecular based diagnostic testing for arboviruses [83]. Technical assistance and interventions of control programmes are routinely offered to countries of concern within the region by organisations, such as, CARPHA [82], WHO, Pan American Health Organization (PAHO) and the United States Agency for International Development (USAID) to lower the incidences of mosquito borne diseases in the country and to reduce the global economic burden of the disease. As recently as, 2016–2019, funding to enhance entomological capacity in the Americas was provided through the Zika AIRS project (ZAP). ZAP was an emergency response programme, funded by USAID, to reduce the spread of the Zika virus within the Americas, minimising its impact. This multi-country initiative actively collaborated with their health authorities and stakeholders to equip national teams with standard protocols, entomological supplies and equipment, surveillance tools and comprehensive laboratory and field training workshops. For the Caribbean countries, Jamaica, Haiti, Dominica Republic and Barbados received donations of an insectary-in-a-box, including equipment to refurbish existing insectaries. Entomological materials for laboratory and field-work, and training for health officials to improve their skills in mosquito surveillance, identification and insecticide susceptibility evaluations were also awarded to Jamaica, Haiti, Dominica Republic, Barbados, Anguilla, Dominica, St. Kitts, St. Lucia and Montserrat [84]. Though aggressive vector control programmes undertaken by ZAP, PASB and PAHO may not be feasible by the governments of Caribbean states, the tools supplied by international programmes provide means to support in country national campaigns, albeit not indefinitely.

#### 3.1.2 Climate model tools

Climate models are being developed under the Economic Commission for Latin America and the Caribbean as prediction tools. In conceptualizing the models, non-climate predictors were included for arboviral diseases. Regression models incorporated the importance of sanitation and water access for the control of dengue, gastroenteritis and leptospirosis. So far, the models performed well in predicting trends in the pattern of the diseases investigated and on all occasions the test of validity demonstrated a mirroring of the trend in the historical disease patterns. The models are being made available as tools within the region [9], the effect of their integration and ease of utilisation as tools of insect disease programmes across the Caribbean is to be determined.

#### 3.1.3 Sterile mosquitoes for SIDS of the Caribbean

Innovative non-insecticidal approaches, example the release of transgenic mosquitoes, to prevent subsequent *A. aegypti* male populations from being generated on mating with wild-type female populations have been used in the Americas. Although the Cayman Islands have claimed success of the programme, long term studies to support the efficacy of the programme are currently not available. In contrast to Brazil, long-term surveillance studies have found that the method was not as successful at preventing F_1_ generation [85]. Other techniques, such as the Sterile Insect Techniques (SIT) have been successfully demonstrated on other vectors of human and veterinary importance in the region [86]. The application of sterile vectors in SIDS presents an attractive alternative method to the use of ineffective pesticides and is environmentally friendly. Challenges that may be incurred under these programmes are the availability of funds required for the initial investment; suitable infrastructure to mount a sustainable programme; availability of qualified personnel in sufficient numbers to conduct the entire procedure and the resilience of the insect to endure packing; transportation to the designated sites and their survival rate in the field.

The success or failure of innovative approaches that include the use of transgenic insects [85] or SIT in the Americas were dependent on the entomological knowledge of the insect, including its behaviour and life-cycle, intensity of field surveillance studies prior to and after release, while practicing an integrative approach to vector management that involved all stakeholders (Dwight Robinson *pers. comm*.).

Other sterility techniques such as the incompatible insect technique (IIT), which exploits the use of *Wolbachia* spp. to infect male mosquitoes, to induce male sterility is at present being explored in non-Caribbean countries [87].

### 3.2 Lessons learnt from the past

The absence of continuous project funding and the non-availability of qualified personnel are the major underlying challenges that affect the lowered success rates of vector control programmes. Today, the strategic approach of integrated vector management as proposed by the WHO describes a holistic methodology for the prevention and management of vector-borne diseases. This strategy involves strengthening disease and vector surveillance within each country, the use of chemical and biological controls, with repetitive investigation on the efficacy of the control measures. Other aspects of integrative management involve community engagement through health promotions and behavioural change, along with the inclusion of multisectoral agents, such as planning and development, water and sanitation, agriculture, education, law and finance. The collective actions of multisectoral agents that impact health and sanitation, and community infrastructure and design, may aid in reducing the cost to achieve effective and sustainable vector management programmes within a country, irrespective of the monitoring and surveillance tools utilised [88].

## 4 Conclusion

Highlighted in this review is that the main insect vector of importance in the English-speaking Caribbean, currently appears to be the mosquito. Numerous studies within the region report reduced efficacy of insecticides against insect vectors, while at the same time reporting an absence of robust medications to treat mosquito-borne illnesses. Additionally, the circulation of more prevalent endemic diseases overshadows the correct diagnosis of less common or novel infectious diseases. Funding for continuous vector control and surveillance along with the continuous training of health authority officials is fiscally unsustainable by Caribbean countries. However, programmes to contain the spread of diseases are routinely executed by external agencies. Practical applications for sustained monitoring and control of neglected insect vector diseases within the Caribbean may benefit from a multisectoral and integrative approach that includes all stakeholders that can be strengthened by the regional support of CARPHA and other governing bodies. Reliable scientific evidence in the English-speaking Caribbean to fill knowledge gaps on arthropod-borne diseases are scarce. The incorporation of research that allows the development of vector management tools, particularly for the arthropod of importance, mosquitoes, as well as routine insect vector management, may alleviate the burden on the local healthcare sector when impacted by known and unknown non-arthropod-borne disease challenges such as climatic disasters or infectious disease pandemics like the COVID-19, which can exhaust meagre resources of the health care system.

## Funding

University of the West Indies, Mona, Jamaica.

## Declaration of Competing Interest

The authors have declared no conflict of interest.

## References

[1] WHO (2021). Control of Neglected Tropical Diseases. https://www.who.int/teams/control-of-neglected-tropical-diseases.

[2] Hotez P. (2008). Holidays in the sun and the caribbean’s forgotten burden of neglected tropical diseases. PLoS Negl. Trop. Dis..

[3] Hotez P.J., Bottazzi M.E., Franco-Paredes C., Ault S.K., P M.R. (2008). The neglected tropical diseases of Latin America and the Caribbean: a review of disease burden and distribution and a roadmap for control and elimination. PLoS Negl. Trop. Dis..

[4] Gondard M., Cabezas-Cruz A., Charles R.A., Vayssier-Taussat M., Albina E., Moutailler S. (2017). Ticks and tick-borne pathogens of the caribbean: current understanding and future directions for more comprehensive surveillance. Front. Cell. Infect. Microbiol..

[5] Gibson K.E., Fitzpatrick D.M., Stone D., N T.P., CNL Macpherson (2016). Vector-borne diseases in the Caribbean: history and current status. CABI Rev..

[6] Chadee D.D., Ward R.A., Novak R.J. (1998). Natural habitats of Aedes Aegypti in the Caribbean--a review. J. Am. Mosq. Control Assoc..

[7] Hugo N., Miller H. (2017). Conflict resolution and recovery in Jamaica: the impact of the zika virus on destination image. Worldwide Hospital. Tour. Themes..

[8] Gammon K. (2014).

[9] Campbell J.D., Taylor M.A., Stephenson T.S., Watson R.A., Whyte F.S. (2011). Future climate of the Caribbean from a regional climate model. Int. J. Climatol..

[10] Petana W.B. (1978).

[11] Lima N.F., Veggiani Aybar C.A., Dantur Juri M.J., Ferreira M.U. (2016). Mansonella ozzardi: a neglected New World filarial nematode. Pathog. Glob. Health..

[12] Post R., Adams Z., Shelly A., Maia-Herzog M., Dias A., Coscaron S. (2003). The morphological discrimination of microfilariae of Onchocerca volvulus from Mansonella ozzardi. Parasitology..

[13] Chadee D.D., Tilluckdharry C.C., Rawlins S.C., Doon R., Nathan M.B. (1995). Mass chemotherapy with diethylcarbamazine for the control of bancroftian filariasis: a twelve-year follow-up in northern trinidad, including observations on Mansonella ozzardi. Am. J.Trop. Med. Hygiene..

[14] Raccurt C., Lowrie R.C., Mcneeley D.F. (1980). Mansonella ozzardi in Haiti. Am. J.Trop. Med. Hygiene..

[15] Couppie P., Clyti E., Sainte-Marie D., Dedet J., Carme B., Pradinaud R. (2004). Disseminated cutaneous leishmaniasis due to Leishmania guyanensis: case of a patient with 425 lesions. Am. J. Trop. Med. Hygiene..

[16] WHO (2021). Vector-Borne Diseases Fact Sheets 2017. https://www.who.int/en/news-room/fact-sheets/detail/vector-borne-diseases.

[17] Desbois N., Pratlong F., Quist D., Dedet J.-P. (2014). Leishmania (Leishmania) martiniquensis n. sp. (Kinetoplastida: Trypanosomatidae), description of the parasite responsible for cutaneous leishmaniasis in Martinique Island (French West Indies). Parasite..

[18] Tikasingh E.S. (1974).

[19] Zeledon R. (1992). Leishmaniasis in the Caribbean Islands a Reviewa. Ann. N. Y. Acad. Sci..

[20] PAHO (2020).

[21] Callahan H.L., Portal I.F., Bensinger S.J., Grogl M. (1996). Leishmania spp: temperature sensitivity of promastigotes in vitro as a model for tropism in vivo. Exp. Parasitol..

[22] Liautaud B., Vignier N., Miossec C., Plumelle Y., Kone M., Delta D. (2015). First case of visceral leishmaniasis caused by leishmania martiniquensis. Am. J.Trop. Med. Hygiene..

[23] Martínez L.P., Rebollo J.A., Luna A.L., Cochero S., Bejarano E.E. (2010). Molecular identification of the parasites causing cutaneous leishmaniasis on the Caribbean coast of Colombia. Parasitol. Res..

[24] González C., Paz A., Ferro C. (2014). Predicted altitudinal shifts and reduced spatial distribution of Leishmania infantum vector species under climate change scenarios in Colombia. Acta Trop..

[25] Khan-Mohammed Z., Adesiyun A.A., Swanston W.H., Chadee D.D. (2005). Frequency and characteristics of selected enteropathogens in fecal and rectal specimens from childhood diarrhea in Trinidad: 1998-2000. Rev. Panam. Salud Publica.

[26] Adesiyun A.A., Kaminjolo J.S., Ngeleka M., Mutani A., Borde G., Harewood W. (2001). A longitudinal study on enteropathogenic infections of livestock in Trinidad. Rev. Soc. Bras. Med. Trop..

[27] Soto E., Griffin M., Verma A., Castillo-Alcala F., Beierschmitt A., Beeler-Marfisi J. (2013). An outbreak of Yersinia enterocolitica in a captive colony of African green monkeys (Chlorocebus aethiops sabaeus) in the Caribbean. Comp. Med..

[28] Wood H., Drebot M.A., Dewailly E., Dillon L., Dimitrova K., Forde M. (2014). Seroprevalence of seven zoonotic pathogens in pregnant women from the Caribbean. Am. J.Trop. Med. Hygiene..

[29] Van Der Kuip E. (1969). Trypanosomiasis cruzi in Aruba and Curaçao. Trop. Geogr. Med..

[30] Gaikhorst G. (1960). The presence of Trypanosoma cruzi on the island of Aruba and its importance to man. Trop. Geogr. Med..

[31] Chikweto A., Kumthekar S., Chawla P., Tiwari K., Perea L., Paterson T. (2014). Seroprevalence of Trypanosoma cruzi in stray and pet dogs in Grenada, West Indies. Trop. Biomed..

[32] Jaramillo R., Bryan J.P., Schur J., Pan A.A. (1997). Prevalence of antibody to Trypanosoma cruzi in three populations in Belize. Am. J. Trop. Med. Hyg..

[33] PAHO (2021). PLISA: Public Health Information Platform for the Americas. https://www.paho.org/data/index.php/en/.

[34] Gubler D.J. (1998). Resurgent vector-borne diseases as a global health problem. Emerg. Infect. Dis..

[35] Rawlins S., Hinds A., Rawlins J. (2008). Malaria and its vectors in the Caribbean: the continuing challenge of the disease forty-five years after eradication from the islands. West Indian Med. J..

[36] Jones M. (2013). A ‘textbook pattern’? Malaria control and eradication in Jamaica, 1910–65. Med. Hist..

[37] Webster-Kerr K., Christie C., Grant A., Chin D., Burrowes H., Clarke K. (2016). Emergence of Zika Virus epidemic and the national response in Jamaica. West Indian Med. J..

[38] Fauci A.S., Morens D.M. (2016). Zika virus in the Americas — yet another arbovirus threat. N. Engl. J. Med..

[39] Quicke Kendra M., Bowen James R., Johnson Erica L., McDonald Circe E., Ma H., O’Neal Justin T. (2016). Zika Virus infects human placental macrophages. Cell Host Microbe.

[40] PAHO/WHO (2020).

[41] Poole-Smith B.K., Hemme R.R., Delorey M., Felix G., Gonzalez A.L., Amador M. (2015). Comparison of vector competence of Aedes mediovittatus and Aedes aegypti for dengue virus: implications for dengue control in the Caribbean. PLoS Negl. Trop. Dis..

[42] Ali I., Mundle M., Anzinger J.J., Sandiford S.L. (2019). Tiger in the sun: a report of Aedes albopictus in Jamaica. Acta Trop..

[43] Francis S., Saavedra-Rodriguez K., Perera R., Paine M., Black W.C., Delgoda R. (2017). Insecticide resistance to permethrin and malathion and associated mechanisms in Aedes aegypti mosquitoes from St. Andrew Jamaica. PLoS One.

[44] Francis S., Crawford J., McKenzie S., Campbell T., Wright D., Hamilton T. (2020). Comparative toxicity of larvicides and growth inhibitors on Aedes aegypti from select areas in Jamaica. R. Soc. Open Sci..

[45] Francis S., Crawford J., McKenzie S., Campbell T., Wright D., Hamilton T. (2020).

[46] Anderson C.R., Downs W.G., Wattley G.H., Ahin N.W., Reese A.A. (1957). Mayaro virus: a new human disease agent. Am. J.Trop. Med. Hygiene..

[47] Talarmin A., Chandler L.J., Kazanji M., de Thoisy B., Debon P., Lelarge J. (1998). Mayaro virus fever in French Guiana: isolation, identification, and seroprevalence. Am. J. Trop. Med. Hygiene..

[48] Hoch A.L., Peterson N.E., LeDuc J.W., Pinheiro F.P. (1981). An outbreak of Mayaro virus disease in Belterra, Brazil. Am. J.Trop. Med. Hygiene..

[49] Marcondes C.B., Fernandes A., Müller G.A. (2006). Mosquitoes (Diptera: Culicidae) near a reservoir in the Western part of the Brazilian State of Santa Catarina. Biota. Neotropica..

[50] Figueiredo L.T.M. (2007). Emergent arboviruses in Brazil. Rev. Soc. Bras. Med. Trop..

[51] Burrell C.J., Howard C.R., Murphy F.A. (2017). Fenner and White’s Medical Virology.

[52] Anderson C.R., Spence L., Downs W.G., Aitken T.H.G. (1961). Oropouche virus: a new human disease agent from Trinidad, West Indies*. Am. J.Trop. Med. Hygiene..

[53] Vasconcelos H.B., Azevedo R.S., Casseb S.M., Nunes-Neto J.P., Chiang J.O., Cantuária P.C. (2009). Oropouche fever epidemic in Northern Brazil: epidemiology and molecular characterization of isolates. J. Clin. Virol..

[54] Aguilar P.V., Barrett A.D., Saeed M.F., Watts D.M., Russell K., Guevara C. (2011). Iquitos virus: a novel reassortant orthobunyavirus associated with human illness in Peru. PLoS Negl. Trop. Dis..

[55] Bastos MdS, Figueiredo L.T.M., Naveca F.G., Monte R.L., Lessa N., Pinto de Figueiredo R.M. (2012). Short report: identification of Oropouche Orthobunyavirus in the cerebrospinal fluid of three patients in the Amazonas, Brazil. Am. J.Trop. Med. Hygiene..

[56] de la Fuente J., Estrada-Pena A., Venzal J.M., Kocan K.M., Sonenshine D.E. (2008). Overview: ticks as vectors of pathogens that cause disease in humans and animals. Front. Biosci..

[57] Barré N., Garris G., Camus E. (1995). Propagation of the tick *Amblyomma variegatum* in the Caribbean. Rev. Sci. Tech. Office Int. Epizooties..

[58] Kelly P., Lucas H., Beati L., Yowell C., Mahan S., Dame J. (2010). Rickettsia africae in Amblyomma variegatum and domestic ruminants on eight Caribbean islands. J. Parasitol..

[59] Kelly P., Fournier P., Parola P., Raoult D. (2003). A survey for spotted fever group Rickettsiae and Ehrlichiae in Amblyomma variergatum from St. Kitts and Nevis. Am. Soc. Trop. Med. Hygiene..

[60] FAO (2019).

[61] Qurollo B.A., Chandrashekar R., Hegarty B.C., Beall M.J., Stillman B.A., Liu J. (2014). A serological survey of tick-borne pathogens in dogs in North America and the Caribbean as assessed by *Anaplasma phagocytophilum*, *A. platys*, *Ehrlichia canis*, *E. chaffeensis*, *E. ewingii*, and *Borrelia burgdorferi* species-specific peptides. Infect. Ecol. Epidemiol..

[62] Perez M., Bodor M., Zhang C., Xiong Q., Rikihisa Y. (2006). Human infection with ehrlichia canis accompanied by clinical signs in Venezuela. Ann. N. Y. Acad. Sci..

[63] Maeda K., Markowitz N., Hawley R.C., Ristic M., Cox D., McDade J.E. (1987). Human infection with Ehrlichia canis, a Leukocytic Rickettsia. N. Engl. J. Med..

[64] Rawlins S.C., Lammie P., Tiwari T., Pons P., Chadee D.D., Oostburg B.F. (2000). Lymphatic filariasis in the Caribbean region: the opportunity for its elimination and certification. Rev. Panam. Salud Publica.

[65] Rawlins S.C., Wan J. (1995). Resistance in some Caribbean populations of Aedes aegypti to several insecticides. J. Am. Mosq. Control Assoc..

[66] Jamaica. WB (2019). World Bank In Jamaica, Overview. https://www.worldbank.org/en/country/jamaica/overview.

[67] Silva L.J., Papaiordanou P.M.O. (2004). Murine (endemic) typhus in Brazil: case report and review. Rev. Inst. Med. Trop. Sao Paulo.

[68] Esposito D.L.A., Fonseca B.A.L. (2017). Will Mayaro virus be responsible for the next outbreak of an arthropod-borne virus in Brazil?. Braz. J. Infect. Dis..

[69] Newman M., McLaren K., Wilson B. (2018). Using the forest-transition model and a proximate cause of deforestation to explain long-term forest cover trends in a Caribbean forest. Land Use Policy.

[70] Reisen W., Fang Y., Martinez V. (2005). Avian host and mosquito (Diptera: Culicidae) vector competence determine the efficiency of West Nile and St. Louis encephalitis virus transmission. J. Med. Entomol..

[71] Stone D., Lyons A., Huang Y.J., Vanlandingham D., Higgs S., Blitvich B. (2018). Serological evidence of widespread exposure of Grenada fruit bats to chikungunya virus. Zoonoses Public Health.

[72] Navarro J.-C., Giambalvo D., Hernandez R., Auguste A.J., Tesh R.B., Weaver S.C. (2016). Isolation of Madre de Dios virus (Orthobunyavirus; Bunyaviridae), an Oropouche virus species reassortant, from a monkey in Venezuela. Am. J. Trop. Med. Hygiene..

[73] Lednicky J., De Rochars V.M.B., Elbadry M., Loeb J., Telisma T., Chavannes S. (2016). Mayaro virus in child with acute febrile illness, Haiti, 2015. Emerg. Infect. Dis..

[74] Brown M., Vickers I., Salas R., Smikle M. (2010). Leptospirosis in suspected cases of dengue in Jamaica, 2002–2007. Trop. Dr..

[75] Cardona-Ospina J.A., Arteaga-Livias K., Villamil-Gómez W.E., Pérez-Díaz C.E., Katterine Bonilla-Aldana D., Mondragon-Cardona Á. (2021). Dengue and COVID-19, overlapping epidemics? An analysis from Colombia. J. Med. Virol..

[76] CARPHA (2020). CARPHA Urges the Caribbean to Increase Efforts to Prevent and Control Mosquito Borne Diseases during the COVID-19 Pandemic. [Internet].

[77] Poveda G. (2020). Concomitant malaria, dengue and COVID-19: an extraordinary challenge for Colombia’s public health system. Curr. Opin. Environ. Sustain..

[78] Amarakoon D., Chen A., Rawlins S., Chadee D.D., Taylor M., Stennett R. (2008). Dengue epidemics in the Caribbean-temperature indices to gauge the potential for onset of dengue. Mitig. Adapt. Strateg. Glob. Chang..

[79] Stennett R. (2008).

[80] Depradine C., Lovell E. (2004). Climatological variables and the incidence of dengue fever in Barbados. Int. J. Environ. Health Res..

[81] Poveda G., Graham N.E., Epstein P.R., Rojas W., Quiñones M.L., Velez I.D. (2000). El Niño and the Southern Oscillation, Multiscale Variability and Global and Regional Impacts.

[82] CARPHA (2020). What We Do. Communicable Diseases. https://carpha.org/What-We-Do/Communicable-Diseases.

[83] CARPHA (2020). Laboratory Testing Services. https://carpha.org/What-We-Do/Laboratory/Laboratory-Testing-Services.

[84] Baranick E. (2019). Zika Program Vector Control. https://www.usaid.gov/sites/default/files/documents/1864/Vector_Fact_Sheet_Feb_2019.pdf:.

[85] Evans B.R., Kotsakiozi P., Costa-da-Silva A.L., Ioshino R.S., Garziera L., Pedrosa M.C. (2019). Transgenic Aedes aegypti mosquitoes transfer genes into a natural population. Sci. Rep..

[86] Dalal P.K., Rathee M., Singh J.K. (2017). Area wide pest management: concept and approaches. Int. J. Curr. Microbiol. App. Sci..

[87] Che-Mendoza A., Martin-Park A., Chávez-Trava J.M., Contreras-Perera Y., Delfín-González H., González-Olvera G. (2021). Abundance and seasonality of Aedes aegypti (Diptera: Culicidae) in two suburban localities of South Mexico, with implications for Wolbachia (Rickettsiales: Rickettsiaceae)-carrying male releases for population suppression. J. Med. Entomol..

[88] CARPHA (2017).

[89] (2021). The Global Health Observatory [Internet]. https://www.who.int/data/gho/data/indicators/indicator-details/GHO/status-of-endemicity.

[90] WHO (2017). Annual Country Reports, 2016.

[91] Aitken T.H.G., Downs W.G., Anderson C.R., Spence L., Casals J. (1960). Mayaro virus isolated from a trinidadian mosquito, Mansonia venezuelensis. Science..

[92] Reed W., Carroll J.S., Agramonte A. (1901). The etiology of yellow fever: an additional note. J. Am. Med. Assoc..

[93] PAHO (2009).

[94] Auguste A.J., Lemey P., Pybus O.G., Suchard M.A., Salas R.A., Adesiyun A.A. (2010). Yellow fever virus maintenance in trinidad and its dispersal throughout the Americas. J. Virol..

[95] De Thoisy B., Dussart P., Kazanji M. (2004). Wild terrestrial rainforest mammals as potential reservoirs for flaviviruses (yellow fever, dengue 2 and St Louis encephalitis viruses) in French Guiana. Trans. R. Soc. Trop. Med. Hyg..

[96] Messina J.P., Brady O.J., Scott T.W., Zou C., Pigott D.M., Duda K.A. (2014). Global spread of dengue virus types: mapping the 70 year history. Trends Microbiol..

[97] Elizondo-Quiroga D., Elizondo-Quiroga A. (2013). West Nile virus and its theories, a big puzzle in Mexico and Latin America. J. Global Infect. Dis..

[98] Downs W.G., Spence L., Aitken T.H., Whitman L. (1961). Cache Valley virus, isolated from a trinidadian mosquito, *Aedes scapularis*. West Indian Med. J..

[99] Harris A.F., Rajatileka S., Ranson H. (2010). Pyrethroid resistance in Aedes aegypti from grand Cayman. Am. J. Trop. Med. Hygiene..

[100] Zwick R. (1964). Susceptibility of two DDT-resistant *Aedes aegypti* strains to DDT 431 and deutero-DDT as larvicides in laboratory tests. Mosquito News..

[101] Marcombe S., Poupardin R., Darriet F., Reynaud S., Bonnet J., Strode C. (2009). Exploring the molecular basis of insecticide resistance in the dengue vector Aedes aegypti: a case study in Martinique Island (French West Indies). BMC Genomics.

